# Case Report: Subfertility and Pregnancy Loss due to Genital Tuberculosis

**DOI:** 10.1007/s42399-023-01492-2

**Published:** 2023-06-15

**Authors:** Y. Stroeken, K. Broekhuijsen, E. Leyten, W. Hermes

**Affiliations:** 1 Present Address: Leids Universitair Medisch Centrum, Leiden, Netherlands; 2 HMC Haaglanden Medisch Centrum, Den Haag, Netherlands; 3 Present Address: Bergman Clinics Hilversum, Hilversum, Netherlands

**Keywords:** Female genital tuberculosis, Subfertility issues, Pregnancy loss, Transplacental exposure, Multi-drug resistant tuberculosis

## Abstract

Tuberculosis (TB) is a disease that primarily affects low and middle income countries (LMICs) but is becoming more relevant in Western countries due to increasing migration from high TB burden countries. It is especially difficult to detect in women with fertility issues as it mimics other more common causes. Delayed diagnosis of TB can result in fallopian tube and endometrial pathology leading to subfertility and pregnancy loss. This case report describes a 34-year-old woman from Ivory Coast who was diagnosed with intrauterine tuberculosis after hysteroscopic evacuation of suspected retained placental tissue following an immature delivery. The patient had a complicated fertility history, including pelvic inflammatory disease and IVF/ICSI procedures, before becoming pregnant at the age of 38. She delivered prematurely at 22 weeks with a retained placenta. A diagnosis of TB was confirmed after pathology revealed granulomatous inflammation, without signs of placental tissue, and further testing confirmed rifampicin-resistant TB. The patient underwent a 15-month course of multi-drug-resistant TB treatment, which postponed her pregnancy wish. The case highlights the challenge of diagnosing genital TB in the female genital tract during subfertility investigations and after a complicated pregnancy in a woman without a history of or symptoms of TB. It underscores the importance of considering TB in the differential diagnosis of subfertility. Screening should be considered in women originating from high endemic countries with unexplained fertility loss and during first trimester screening.

## Introduction

Tuberculosis is a global infectious disease with a high prevalence worldwide and a high burden in terms of morbidity and mortality [1]. While TB is uncommon in the Netherlands, particularly in gynecological practice, it remains prevalent in low and middle income countries [2]. It is especially difficult to detect TB in women with fertility issues as it mimics other more common causes. Failure to detect TB early can result in fallopian tube and endometrial pathology leading to subfertility and pregnancy loss [3]. In this case report, we describe a 34-year-old woman with intrauterine TB that was diagnosed after hysteroscopic evacuation of suspected retained placental tissue following an immature delivery.

## Case presentation

### Fertility history

In 2014, a 34-year-old woman from Ivory Coast visited our clinic with signs of pelvic inflammatory disease (PID) and a positive chlamydia antibody test. Ultrasound imaging revealed large abscesses, and blood tests showed elevated infection parameters. She underwent laparoscopic surgery; during which, the left obstructed tube was drained and the right tube was removed due to pyosalpinx. A purulent fluid was drained from the pyosalpinx and sent for microbiologic examination. A general culture was negative, and PCR for Chlamydia was also negative. Recent cervical swabs were also negative for sexually-transmitted infections. The removed tube was send for pathology, and the histology sample showed fibrotic tissue with small granuloma’s and mild necrosis. Further investigation with Ziehl–Neelsen, Periodic Acid Shift (PAS), and Grocott was performed and no signs of yeast, actinomyces, or tuberculosis could be found.

After the operation, the patient was referred to our fertility clinic for a full work-up, but did not follow through with the appointment until 2018. History revealed that she had one child with another partner, fourteen years prior. Hormonal status was normal, and ultrasound showed a normal uterus with a 3.5 mm endometrial thickness and a left ovary with a small cyst (33 × 26 mm) containing cloudy fluid, which was considered to be an endometriosis cyst. The patient and her partner underwent in vitro fertilization (IVF). IVF with embryo-transfer was the choice of treatment. Four embryos could be grown and two embryos of, however, low quality, were transferred into the uterus. Unfortunately, the patient did not become pregnant. Due to COVID-19, further treatment was postponed until 2022, when the patient and partner started an intracytoplasmic sperm injection (ICSI) procedure and the patient became pregnant.

### Pregnancy

At the time of pregnancy, she was 38 years old. At 10 weeks gestation, a normal ultrasound was performed without any anomalies. However, at 18 weeks gestation, the patient experienced vaginal bleeding, and an extensive ultrasound revealed a sub-placental hematoma. The fetus did not have any structural abnormalities, and the cervical length was 27 mm. Cervical swabs for gonorrhea, chlamydia, bacterial vaginosis, and yeast infections, as well as a PAP smear showed no anomalies. Unfortunately, she returned to the hospital after a new episode of bleeding and cramps and delivered immaturely at 22 weeks gestation.

**Fig. 1:**
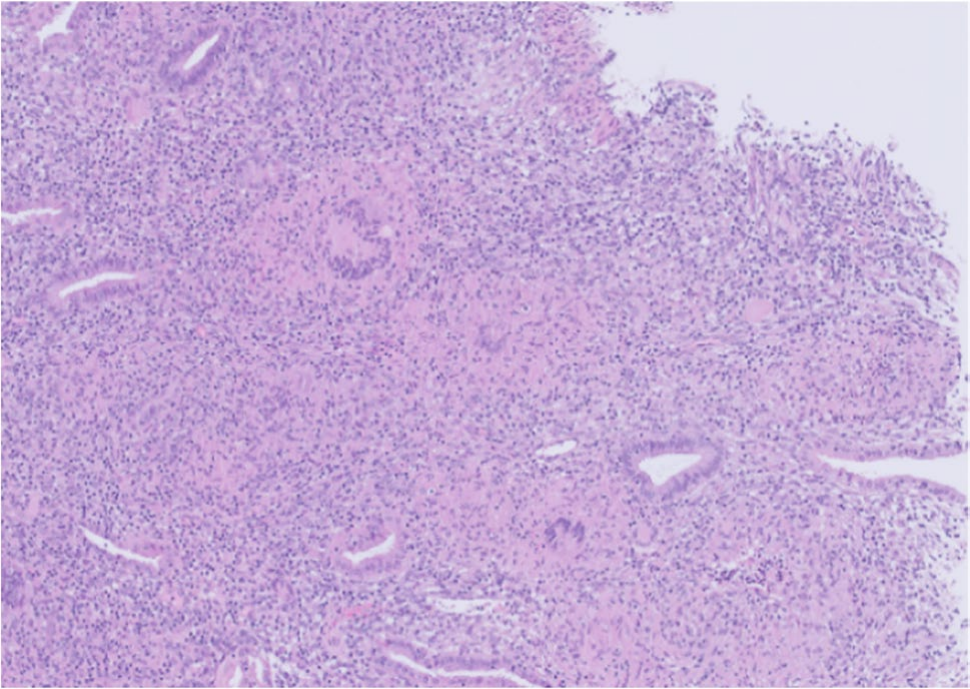
Granulomata and giant cells in endometrial tissue

The delivery was complicated by a retained placenta, which was evacuated via vacuum curettage. Afterwards, the placenta was sent to the pathologist for further examination. The pathology report showed a necrotic granulomatous inflammation among others (Fig. 1).

During a routine postpartum check-up, the patient reported continuous irregular blood loss and lower abdominal pain. The ultrasound showed an intrauterine homogeneous structure of 13 mm, suspicious for a retained placental tissue. We scheduled her for a hysteroscopy to remove the suspected placental rest.

During this procedure, a large amount of thick, yellow, fibrotic tissue was visualized, which could only be partially resected. The procedure was ended due to reaching maximum fluid loss. The pathology report revealed the same granulomatous inflammation, without signs of placental tissue. After consultation with the pathologist, microbiologist, and internal medicine-interstitial lung disease specialist, the following differential diagnosis was drawn up; TB, actinomyces species, auto-immune vasculitis, and auto-immune diseases. A diagnostic work-up was planned accordingly. More tissue was collected by endometrium aspiration technique, which was send for pathology and culture purposes to examine for actinomyces and TB. The Ziehl–Neelsen (ZN) and auramine testing results were negative.

In addition, blood was taken for IGRA (QuantiFERON-TB) and a wider panel of tests on IL2-R, ANCAs, ENA, and antibodies.

A recent X-ray of the lungs taken because of shortness of breath by the general practitioner showed no signs of lung diseases. The QFT-TB was positive and culture results of the extracted tissue came back positive for Mycobacterium tuberculosis and rifampicin resistant.

Accordingly, the patient was referred to an internal medicine infectious disease specialist for further diagnosis, treatment, and follow-up. An extended history was taken with the patient, but she could not recall any periods of coughing, shortness of breath, or night sweats. A CT scan was performed, which showed a suspected tuberculosis granuloma in the left kidney, this was confirmed by a PET-CT and a biopsy. No other sources containing TB were found.

The Mycobacterium tuberculosis turned out to be isoniazid and rifampicin resistant. Therefore, we referred the patient to University Medical Center Groningen (UMCG), one of the two tertiary specialized centers in the Netherlands. She was discharged after she started with her treatment for multi-drug resistant tuberculosis for a total duration of 15 months, which is partly teratogenic. For this reason, she had to postpone her pregnancy wish. The patient conceived again in 2023, but unfortunately, this pregnancy resulted in a miscarriage at eight weeks gestation.

This case report shows the difficulty of diagnosing extrapulmonary TB in a woman without history of and/or symptoms of tuberculosis in a western country where TB is uncommon.

## Background

Active tuberculosis, caused by the Mycobacterium tuberculosis bacillus, has the highest mortality rate of all infectious diseases worldwide [1]. These Mycobacteria are mostly spread through aerosols but can also be transmitted vertically from mother to fetus or neonate. It is estimated that around 25% of the world population is or has been infected with TB, with most cases in low and middle income countries (LMIC) [1]. The risk of developing active TB rises with previously acquired HIV infection and increasing drug resistance.

While a reduction in incidence and mortality of 9% and 14%, respectively, worldwide was achieved between 2015 and 2019, it still remains a major problem. [1]

In the Netherlands, most people (about 75%) with active TB are first or second-generation immigrants. Other risk groups include homeless people and people with compromised immunity, of which 3% are caused by HIV [2]. In all of these groups, proactive detection of TB infections is recommended. [2]

For all subtypes—pulmonary or extrapulmonary disease—one of the important first-line drugs is rifampicin, in combination with others [4, 5]. However, in 2017, 24% of all new cases and up to 70% of the previously treated patients worldwide were found to be rifampicin resistant.

### Pathophysiology

As stated above, TB is generally airborne; and in 79–87% of the cases with an active TB infection, the lungs are involved. [6]

The majority of the patients who are infected with TB clear the Mycobacterium after a primary infection. In a fair part of the patients, spreading of disease occurs. A latent phase of the disease is entered in 5–10% of the cases, with reactivation occurring later in life. [6]

### Extrapulmonary disease

It is estimated that about 15% of all incident cases are extrapulmonary TB [6]. This could be developed after a progressive primary disease or reactivation of latent disease. Most common sites for infection are lymph nodes, pleura effusion, and bone and joints, but it is also described in the urogenital system, nervous system, abdomen, and pericard [7]. Furthermore, extrapulmonary disease is seen in about 50% of the patients affected with AIDS. Fortunately, only active pulmonary disease can spread through air to others, so isolation measures are not needed in case of suspected extrapulmonary TB.

### Effect on fertility and pregnancy

Genital tuberculosis is usually secondary to hematogenic spread from a primary focus, most commonly the lungs. In rare cases, primary infection from intercourse with a man with infection of the penis or epididymis is described [8]. Fertility can be impaired by extrapulmonary TB involving the genital tract. Genital involvement occurs in about 9% of women with TB. The fallopian tubes are most commonly involved showing hydro- or pyosalpinx and dense adhesions, thereby causing subfertility and a higher risk of ectopic pregnancy. Infection of the endometrium is second most common side for genital TB. Furthermore, infection of the uterus, ovaries, and peritoneum has been described. [9, 10]

To our knowledge, there has only been one case in which an infection of the chorion with Mycobacterium tuberculosis was confirmed and reported [11]. In some other cases, a granulomatosis reaction has been described in removed placental tissue, but tests for TB were not available. [12, 13]

The effect of pregnancy on the risk of reactivation of latent TB does not appear to be increased, and risk factors for developing TB are similar to the general population. However, a higher incidence of TB is reported in the postnatal period as a result of immunologic changes. [14]

Conversely, neonatal and maternal outcomes seem to be impaired by TB. A meta-analysis of thirteen studies from Sobhy et al. (2017) found that maternal morbidity was three times higher in the TB group, with significantly increased incidence of anemia, cesarean section, and miscarriage. Active disease can spread vertically through the placenta via bloodstream or lymphatics or be transmitted during delivery via amniotic fluid.

The risk of perinatal death and preterm birth was four times and 1.6 times higher in the TB group compared to the control group, respectively. The risk of congenital anomalies and small for gestational age did not differ significantly [15]. Other studies did show rates of preterm birth up to five times higher and did find a fetal growth restriction up to three times higher. [16, 17]

### Diagnosis and treatment of extrapulmonary TB

Clinically, female genital tuberculosis either presents with subfertility, abdominal pain, or menstrual disorders [18]. Imaging often shows non-specific signs on ultrasound or hysteroscopy such as fallopian tube dilatation, stenosis, nodular scarring, and hydro- or pyosalpinx. When the endometrium is affected, the ultrasound can show distended endometrium with heterogenous material or calcifications. These signs are quite common and a specific in subfertility examinations and could be caused by multiple infections, endometriosis, or previous intra-abdominal or intra-uterine operations [18]. Laparoscopic surgery could show more specific signs such as tubercles or caseous granuloma on the fallopian tubes or peritoneum. Biopsy of these abnormalities in combination with Tuberculin/Mantoux or Interferon Gamma Release Assay (IGRA) or microbial tests from the specimen such as PCR could diagnose tuberculosis more accurate and faster. [19]

Female genital tuberculosis should be treated with a four-drug regimen due to the high relapse rates and common drug-resistant types of tuberculosis. This regimen consists of a combination of bactericidal drugs (isoniazid, rifampicin, pyrazinamide, and streptomycin) and bacteriostatic drugs (ethambutol or ethionamide) [7] for a period of 9–12 months depending on the resistance pattern [2, 8]. This could be supplemented with surgery in case of large abscesses or blockage of the fallopian tubes. [20]

## Discussion and recommendations

We presented a case of intrauterine TB discovered after hysteroscopic resection of what was assumed to be retained placental tissue. In retrospect, our patient’s secondary subfertility and recent immature delivery were probably caused by chronic TB in the endometrium and fallopian tubes. The patient was likely infected after her first pregnancy. This case highlights the difficulties in detecting extrapulmonary TB in patients without clear pulmonary symptoms, particularly in non-endemic areas where the index of suspicion is low.

Through this case report, we aim to raise awareness about extrapulmonary TB and its various manifestations, such as the female genital tract.

Another lesson learned from this case is that a wide range of alternative tests had been done, which may have been unnecessary if different diagnostics had been done in the first place. This patient originated from Ivory Coast, a high endemic country for TB. Subfertility caused by fallopian tube blockage and early miscarriages due to endometrial involvement are some of the most common symptoms of female genital tuberculosis. The negative ZN- and auramine staining results from both operations, brought doubts about the most probable diagnosis of TB. However, the specimen should contain at least 10 [6] colony forming units (CFU)/mL to count as a positive smear for ZN staining. Additionally, auramine staining does not differentiate different types of mycobacteria from each other [3]. Therefore, histopathology examination in combination with PCR samples should be the gold standard in high-resource countries.

Although morbidity rates in pregnant women with TB are three times higher, screening for TB in all women with impaired fertility or during first-trimester screening is not beneficial, as there are only 4.4 cases of TB per 100,000 residents with TB in the Netherlands. [2]

However, with the rising trends in immigration and the flattening decrease in TB incidence, we suggest screening for TB during preconceptional or first-trimester screening in women with risk factors, such as immigrants from TB high-endemic regions, HIV, and/or unknown cause of subfertility. QuantiFERON-TB test is the preferred method of screening. If this test is positive, additional screening with biopsy or PCR sampling from fallopian tubes or endometrium should be performed.

Early detection of the disease will allow for the prevention of morbidity and mortality in women and their fetuses.

## Data Availability

N/A.

## Code availability

N/A.
